# Combined inspiratory muscle and lower limb resistance training enhances cardiopulmonary function in military personnel

**DOI:** 10.3389/fphys.2026.1766008

**Published:** 2026-03-11

**Authors:** Xiaolong Ma, Houyuan Zhu, Jingqi Tao, Mingming Jian, Shaoqi Huang

**Affiliations:** 1 PLA Army Engineering University, Shijiazhuang, China; 2 Jimei University, Xiamen, China

**Keywords:** cardiopulmonary function, inspiratory muscle training (IMT), military personnel, resistance training, VO2max

## Abstract

**Objective:**

This study aimed to investigate the effects of combining inspiratory muscle resistance training (IMT) with lower limb resistance training on cardiopulmonary function and respiratory muscle strength in military academy cadets.

**Methods:**

Sixty non-commissioned officer cadets (aged 18–22 years) from the Army Engineering University were recruited and randomly divided into an experimental group (n = 30) and a control group (n = 30). Both groups underwent a 12-week intervention including baseline aerobic running. The control group performed IMT alone, while the experimental group performed IMT combined with specific lower limb resistance exercises. Respiratory muscle strength (MIP, MEP), cardiopulmonary exercise testing (CPET) metrics, and static pulmonary function were assessed pre- and post-intervention.

**Results:**

Following the 12-week intervention, both groups showed significant improvements in respiratory muscle strength and cardiopulmonary function. Notably, the experimental group exhibited significantly greater enhancements in maximal oxygen uptake (VO_2_max) and maximal inspiratory pressure (MIP) compared to the control group (P < 0.05). No significant changes were observed in static pulmonary function indices (SVC, FVC, FEV_1_, MVV) for either group, likely due to the participants’ high baseline fitness.

**Conclusion:**

While IMT is effective for improving respiratory efficiency, its combination with lower limb resistance training yields synergistic effects, leading to superior improvements in VO_2_max and inspiratory muscle strength. This combined modality offers a scientifically grounded strategy to optimize the physical readiness and combat capability of military personnel.

## 1 Introduction

Military physical training occupies a pivotal role in the daily training regimen of armed forces, with its core objective of improving or maintaining the physical fitness of military personnel. This ensures that service members can adapt to complex, high-risk, and high-intensity combat environments with optimal physical status and sufficient energy reserves (Heinrich et al., 2012). Among the multiple factors influencing military personnel’s physical fitness and combat capability, cardiopulmonary function stands out as a critical determinant. Previous studies have demonstrated that elite special forces units exhibit significantly improved endurance performance following high-intensity respiratory muscle training (RMT) (Sperlich et al., 2009; Kraemer et al., 2012). A well-functioning cardiopulmonary system enhances aerobic endurance and physical performance, which directly translates to improved combat effectiveness in operational settings. Recent research has further highlighted the systemic impact of respiratory muscle function. Studies have shown that inspiratory muscle fatigue can impair not only autonomic cardiac control (Ladriñán-Maestro et al., 2024a) but also lower limb muscle oxygenation and vertical jump performance (Ladriñán-Maestro et al., 2024b), thereby underscoring the critical role of respiratory muscles in overall physical fitness.

In recent years, the integration of multiple training modalities to enhance military physical fitness has emerged as a research hotspot. Among these modalities, inspiratory muscle training (IMT) and lower limb resistance training have garnered considerable attention due to their proven efficacy in improving cardiopulmonary function and muscle strength, respectively (Martins et al., 2019). Respiratory muscles refer to the muscle groups involved in the breathing process. During high-intensity or prolonged exercise, respiratory muscle fatigue increases sympathetic nerve activity and induces vasoconstriction in working limbs. This impairs skeletal muscle blood perfusion, ultimately reducing exercise performance (Pessoa et al., 2014). Therefore, targeted respiratory muscle training is essential to delay fatigue and optimize exercise capacity.

Maximal inspiratory pressure (MIP) and maximal expiratory pressure (MEP) are well-validated, non-invasive indices for evaluating respiratory muscle strength, with MIP also serving as a rapid measure of diaphragmatic strength (Sachs et al., 2009). IMT, typically performed using a respiratory resistance trainer, improves cardiopulmonary function by enhancing the strength and endurance of inspiratory muscles (Illi et al., 2012). Clinical and sports science research has consistently shown that IMT increases vital capacity, improves respiratory efficiency, and reduces perceived dyspnea (Riera et al., 2001). Concurrently, lower limb resistance training—a classic strength training modality—not only enhances lower limb muscle strength but also indirectly improves cardiopulmonary function by increasing cardiopulmonary load during exercise (Clark et al., 2008).

Despite the extensive body of research supporting the individual benefits of IMT and lower limb resistance training, studies exploring their combined effects remain relatively scarce. Combined training is a common practice in exercise physiology, and existing evidence suggests that integrating different training modalities can elicit synergistic effects, leading to more comprehensive improvements in physical function (Ammar et al., 2020; Zaenker et al., 2016; Yu et al., 2023; Junior et al., 2017; Simão et al., 2011). For instance.

Combined aerobic-anaerobic training: Integrates aerobic exercises (e.g., running, swimming) with anaerobic exercises (e.g., weight training, high-intensity interval training) to enhance cardiopulmonary endurance, muscle strength, metabolic adaptability, and athletic performance (Ammar et al., 2020; Zaenker et al., 2016).

Combined respiratory-core stability training: Focuses on strengthening core muscle groups to improve balance, movement control, and reduce injury risk while simultaneously enhancing respiratory function (Yu et al., 2023).

Combined flexibility-strength training: Combines flexibility exercises (e.g., yoga, stretching) with resistance training to improve muscle flexibility, extensibility, strength, and power, thereby significantly boosting athletic performance (Junior et al., 2017; Simão et al., 2011).

Notably, recent studies have confirmed that improved respiratory muscle function can enhance the performance of middle- and long-distance runners (Chang et al., 2021). Given this, combining IMT with lower limb resistance training holds significant potential for improving the cardiopulmonary function, inspiratory muscle strength, and ultimately the combat capability of military personnel.

The present study was designed to systematically investigate the effects of combined IMT and lower limb resistance training on cardiopulmonary function and respiratory muscle strength in military academy cadets. By analyzing the comprehensive effects of this combined training modality, we aim to provide a scientific basis for military physical training programs and offer new insights into enhancing the overall physical fitness of military personnel.

## 2 Participants and methods

### 2.1 Research participants

Sixty non-commissioned officer cadets from the Shijiazhuang Campus of the Army Engineering University were recruited as study participants. All cadets were aged 18–22 years, had accumulated years of prior military training experience, and were in active service. “The study protocols involving human participants were reviewed and approved by the training department of the PLA Army Engineering University. In accordance with the Declaration of Helsinki and the regulations for military physical training, formal ethical approval was waived as the training intervention was integrated into the cadets’ standard daily physical training regimen. Written informed consent was obtained from all participants prior to the study.

### 2.2 Research methods

#### 2.2.1 Experimental protocol

Before initiating any tests or training, all participants completed a baseline questionnaire designed in accordance with the Chinese Expert Consensus on the Assessment and Monitoring of Exercise-Related Cardiovascular Event Risks (Zhao et al., 2022). This questionnaire collected information on medical history, exercise habits, and family genetic history to stratify participants by cardiovascular risk. Individuals classified as high-risk were excluded from the study to ensure safety.

**FIGURE 1:**
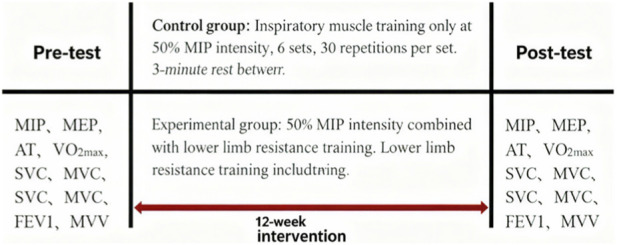
Schematic of the experimental intervention protocol.

Both groups underwent baseline (pre-training) and post-training assessments, including respiratory muscle strength tests, cardiopulmonary exercise testing (CPET), and static pulmonary function tests. The training intervention lasted for 12 weeks (one academic semester), during which no other forms of structured training were permitted to avoid confounding effects. The detailed experimental protocol is illustrated in Figure 1.

Control group: IMT alone at 50% of individual MIP, consisting of 6 sets of 30 repetitions per session, with 3 min of rest between sets.

Experimental group: Combined training of IMT (50% of individual MIP) and lower limb resistance training. Lower limb resistance training included two exercises (squats and single-leg squats), with 3 sets per exercise (6 sets total) and 20 repetitions per set, plus 3 min of rest between sets.

Pre-training assessments: MIP, MEP, anaerobic threshold (AT), VO_2_max, slow vital capacity (SVC), forced vital capacity (FVC), forced expiratory volume in 1 s (FEV_1_), maximal voluntary ventilation (MVV).

Post-training assessments: Same as pre-training assessments.

Intervention duration: 12 weeks.

#### 2.2.2 Respiratory muscle strength test

MIP and MEP were used as primary indices to evaluate respiratory muscle strength (Pessoa et al., 2014). A respiratory muscle assessment and training device (compliant with American Thoracic Society/European Respiratory Society (ATS/ERS) standards, Model JL-REX01F) was employed to measure MIP and MEP. All measurements were performed by a trained operator following a standardized protocol to ensure consistency.

Participants were seated upright on the front half of a chair, with nasal passages occluded using a nose clip, feet shoulder-width apart on the floor, and torso kept straight. For MIP measurement: Participants exhaled completely to residual volume, then sealed their lips tightly around the mouthpiece and inhaled maximally for approximately 3 s. The operator provided verbal encouragement to ensure maximal effort. For MEP measurement: Participants inhaled completely to total lung capacity, then sealed their lips around the mouthpiece and exhaled maximally for approximately 3 s, with verbal encouragement from the operator.

**FIGURE 2:**
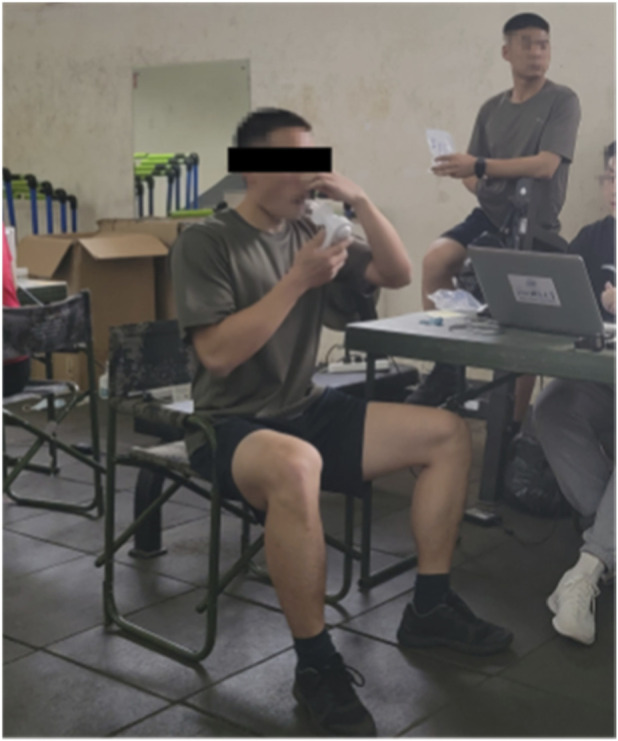
Schematic of the respiratory muscle strength test procedure.

Due to time constraints, participants were allowed 2 maximal attempts for each test, with a 60-s rest interval between attempts. Attempts were discarded if improper technique was observed (e.g., excessive use of buccinator muscles). The measurement process is illustrated in Figure 2.

#### 2.2.3 Cardiopulmonary exercise testing (CPET)

Prior to CPET, participants received a detailed explanation of the test procedure, potential risks, and safety precautions. CPET was conducted using a cardiopulmonary exercise testing system (Hanya, China, Model Smax58ce-sp). The test protocol was as follows:

Warm-up phase: Participants ran on a treadmill at 7 km/h for 3 min to acclimate to the equipment and exercise intensity.

Incremental exercise phase: After warm-up, treadmill speed was increased linearly by 1 km/h per minute, with a constant incline of 1% to simulate wind resistance.

During the test, a 10-lead ambulatory electrocardiography (ECG) and ambulatory blood pressure monitoring system (Labtech, Hungary, Model EC-12S) were used to continuously monitor cardiac activity and blood pressure. Additionally, participants rated their perceived exertion using the Rating of Perceived Exertion (RPE) scale (Beaver et al., 1986). These monitoring measures ensured the safety of participants throughout the test.

The test was terminated when participants reached maximal oxygen uptake (VO_2_max), which was defined by the presence of any 2 of the following criteria:

1. Heart rate reached the age-predicted maximal heart rate (220 − age).
2. Respiratory exchange ratio (RER) exceeded 1.10.
3. Oxygen uptake plateaued or decreased despite increasing exercise intensity.
4. RPE ≥17 (indicating maximal voluntary effort).

Upon test termination, VO_2_max and relative VO_2_max (VO_2_max/kg, mL/min/kg) were recorded. The anaerobic threshold (AT) was determined using the V-slope method, which identifies the inflection point in the relationship between oxygen uptake (VO_2_) and carbon dioxide production (VCO_2_) (Beaver et al., 1986). The ratio of AT to VO_2_max (AT/VO_2_max, expressed as a percentage) was calculated to indirectly assess aerobic capacity.

#### 2.2.4 Static pulmonary function tests

Participants were seated upright on a chair, with a face mask securely fitted. Static pulmonary function tests were performed using the same cardiopulmonary exercise testing system (Hanya, China, Model Smax58ce-sp). Participants were instructed on the correct breathing techniques (inhalation, breath-holding, and exhalation) to ensure accurate measurements. The tests included:

1. Slow Vital Capacity (SVC): Participants performed 4–5 normal breaths, then inhaled slowly and maximally to total lung capacity, followed by a slow, continuous exhalation until no further air could be expelled. SVC was recorded as the maximum volume of air exhaled.
2. Forced Vital Capacity (FVC): After 4–5 normal breaths, participants inhaled rapidly to total lung capacity, then exhaled as forcefully and rapidly as possible for 6 s. FVC was recorded as the total volume of air exhaled during this forced maneuver.
3. Forced Expiratory Volume in 1 Second (FEV_1_): This was defined as the volume of air exhaled in the first second of the forced exhalation maneuver described for FVC.
4. Maximal Voluntary Ventilation (MVV): Participants performed 4–5 normal breaths, then completed rapid, deep breaths (1 inhalation +1 exhalation per cycle) for 12 s. The optimal 6–12 s segment was used to calculate MVV (extrapolated to 1 min).

#### 2.2.5 Training protocol

Participants were randomly assigned to the experimental group or control group using a computer-generated random number sequence. Baseline differences between the two groups were analyzed using independent samples t-tests (Table 1). No significant differences were observed in any of the measured indices before training, indicating that the two groups were comparable at baseline.

**TABLE 1:** Baseline measurements of the experimental and control groups.

Index	Experimental group (n = 30)	Control group (n = 30)	P
AT (%VO_2_max)	85.11 ± 8.27	84.51 ± 6.88	0.825
VO_2_max/kg (mL/min/kg)	58.51 ± 7.23	59.02 ± 6.75	0.865
SVC(L)	4.81 ± 0.65	4.79 ± 0.54	0.826
FVC(L)	4.95 ± 0.55	4.95 ± 0.68	0.384
FEV1(L)	4.01 ± 0.35	3.98 ± 0.29	0.409
MVV(L)	122.65 ± 11.57	123.86 ± 10.85	0.321
MIP(cmH2O)	155.56 ± 22.36	156.05 ± 21.45	0.369
MEP (cmH2O)	158.58 ± 17.13	158.56 ± 22.02	0.222

Numerous studies have confirmed that IMT at 50% of MIP effectively improves respiratory muscle function, whereas training at 15% of MIP yields no significant benefits (Archiza et al., 2018; Caine and Mcconnell, 1998; Volianitis et al., 2001; Cunha et al., 2019; Liu, 2007). Based on these findings and established training protocols (Volianitis et al., 2001; Shuai et al., 2022), the present study adopted the following training regimen:

Training frequency: 3 sessions per week for 12 weeks.

Aerobic exercise component: All participants completed a 5 km run at 90% of their individual anaerobic threshold (determined via pre-training CPET). Heart rate was monitored throughout the run using a wristwatch to ensure exercise intensity remained within the target range.

IMT equipment: A POWER®breathe device (POWER®breathe, Southam, UK) was used for IMT. This device is a commercially available linear resistance trainer that generates resistance via a spring-loaded system or electronic valve, with proven efficacy in inspiratory muscle training (Nepomuceno Júnior et al., 2016).

**FIGURE 3:**
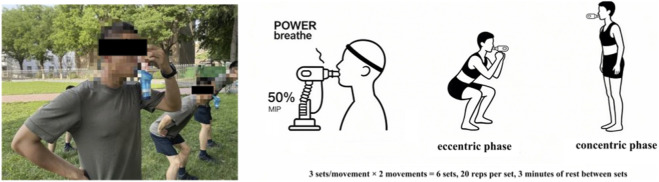
Combined training of inspiratory muscle resistance and lower limb resistance.

Experimental group: Combined training of IMT (50% of MIP) and lower limb resistance training. Lower limb resistance training included two exercises: squats and single-leg squats. Breathing technique was standardized: exhalation during the eccentric phase (squatting) and inhalation during the concentric phase (standing up). Each exercise was performed for 3 sets (6 sets total), with 20 repetitions per set and 3 min of rest between sets. The training procedure is illustrated in Figure 3.

Control group: IMT alone at 50% of MIP, consisting of 6 sets of 30 repetitions per session, with 3 min of rest between sets.

During IMT, participants inhaled maximally from residual volume against the preset resistance until no further chest expansion was possible. Exhalation was performed slowly and gently to avoid hyperventilation. Inspiratory pressure was monitored continuously during each breath to ensure adherence to the target training intensity. Participants were instructed to maintain a steady breathing rhythm to prevent excessive increases in respiratory rate or tidal volume, which could lead to hyperventilation.

Each training session followed a fixed sequence: IMT first, followed by the 5 km run. After each session, participants recorded their RPE to monitor subjective exercise intensity. Each training session followed a fixed sequence: IMT first, followed by the 5 km run. After each session, participants recorded their RPE to monitor subjective exercise intensity.

### 2.3 Data processing and statistical analysis

All data are presented as mean ± standard error of the mean (Mean ± SEM). The normality of the data distribution was assessed using the Shapiro-Wilk test. Since the data followed a normal distribution, a two-way repeated measures analysis of variance (ANOVA) was conducted to determine the effects of Time (Pre-training vs. Post-training) and Group (Experimental vs. Control), as well as the Time × Group interaction. When a significant interaction was observed, simple main effects were analyzed using Bonferroni *post hoc* adjustments. Statistical analyses were performed using SPSS 22.0 software (IBM Corp., Armonk, NY, United States). A P-value <0.05 was considered statistically significant.

## 3 Results

### 3.1 Respiratory muscle strength

**FIGURE 4:**
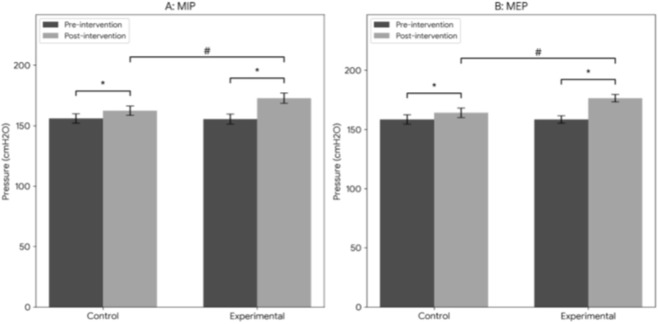
Changes in (MIP) and (MEP) after 12-week intervention. Data are presented as mean ± SEM. *P < 0.05 indicates a significant difference compared to baseline within each group; #P < 0.05 indicates a significant difference between the experimental group and the control group post-training.

As shown in Figure 4, after 12 weeks of intervention, both the control group and the experimental group exhibited a statistically significant increase in MIP and MEP (P < 0.01). Furthermore, the experimental group had significantly higher MIP and MEP values than the control group post-training (P < 0.05), indicating that the combined training regimen was more effective in enhancing respiratory muscle strength.

### 3.2 Cardiopulmonary exercise test outcomes

**FIGURE 5:**
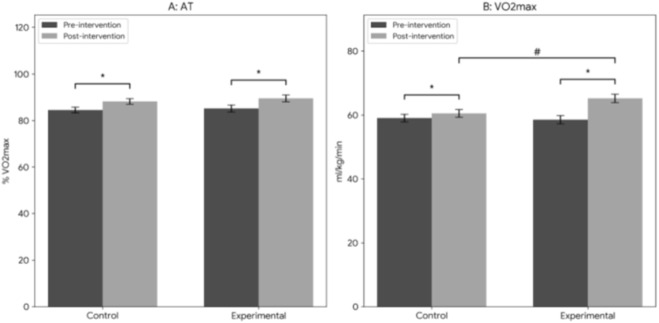
Changes after 12-week intervention (AT) and (VO_2_max). Data are presented as mean ± SEM. *P < 0.05 indicates a significant difference compared to baseline within each group; #P < 0.05 indicates a significant difference between the experimental group and the control group post-training.

Figure 5 illustrates the changes in AT and VO_2_max before and after training. After 12 weeks of intervention, both groups showed a statistically significant increase in AT and VO_2_max compared to baseline (P < 0.01). Importantly, the experimental group had a significantly higher VO_2_max than the control group post-training (P < 0.05), demonstrating that combined training was more effective in improving maximal aerobic capacity. No significant between-group difference was observed in AT post-training (P > 0.05).

Repeated measures ANOVA revealed a significant Group × Time interaction for VO2max (F_1.58_ = 5.24, P < 0.05). The experimental group showed significantly greater improvement compared to the control group.

### 3.3 Static pulmonary function

**FIGURE 6:**
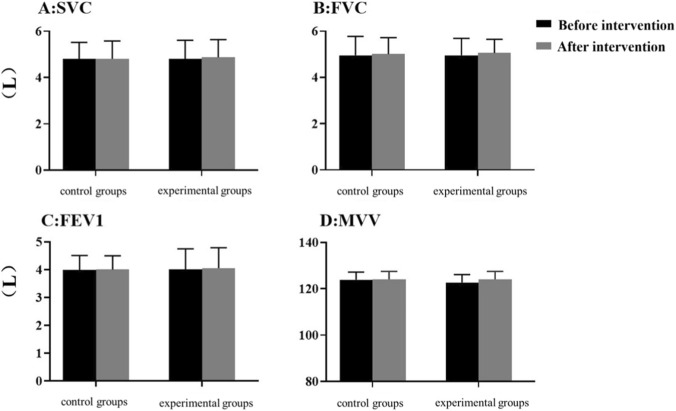
Changes after 12 weeks of intervention (SVC, FVC, FEV_1_, MVV) Data are presented as mean ± SEM. No significant differences were observed between groups or compared to baseline (P > 0.05).

As shown in Figure 6, no statistically significant changes in SVC, FVC, FEV_1_, or MVV were observed in either group after the 12-week intervention (P > 0.05). Additionally, there were no significant differences in these indices between the experimental group and the control group post-training (P > 0.05), suggesting that neither IMT alone nor combined training had a significant impact on static pulmonary function in this population.

## 4 Discussion

The present study systematically evaluated the effects of combined IMT and lower limb resistance training on cardiopulmonary function and respiratory muscle strength in military academy cadets. The results confirmed the efficacy of this combined training modality: both groups showed significant improvements in VO_2_max and MIP post-training, with the experimental group (combined training) demonstrating more pronounced enhancements than the control group (IMT alone). These findings indicate that IMT can effectively improve VO_2_max, AT, MIP, and MEP in military personnel, and combining IMT with lower limb resistance training yields additional benefits for inspiratory muscle function.

### 4.1 Historical context of respiratory muscle research

Early research on respiratory muscles primarily focused on clinical populations. For example, Hautmann et al. defined the lower limit of normal reference values for MIP in healthy adults aged 18–82 years with normal pulmonary function, providing a critical reference for pulmonary function laboratories to evaluate MIP in both young and elderly patients (Hautmann et al., 2000). Subsequent studies extended this work to pediatric populations: a study on maximal respiratory pressure in healthy children found that boys had higher maximal respiratory pressure values than girls, and these values increased with age (Marcelino et al., 2022).

Beyond pulmonary function, researchers have explored correlations between MIP and other physiological indices. A 2016 study reported a strong positive correlation between MIP and handgrip strength in healthy young and middle-aged adults, suggesting that handgrip strength could serve as an indirect indicator of inspiratory muscle strength (particularly diaphragmatic strength). This study also highlighted that respiratory diseases or pathological conditions can significantly reduce MIP (Raab et al., 2016).

### 4.2 Respiratory muscle function in sports and military settings

In recent years, sports science has increasingly focused on respiratory muscle function, particularly its role in endurance performance. MIP and MEP—as key indices of respiratory muscle strength—are critical for endurance exercise, where sustained oxygen delivery is essential for maintaining performance. Higher MIP and MEP have also been associated with improved running speed and power output in athletes (Kilding et al., 2010; Romer and Polkey, 1985). This is because strong respiratory muscles can generate greater airflow and lung volumes, allowing athletes to maintain normal respiratory rate and depth even at high exercise intensities (Kilding et al., 2010).

Respiratory muscle strength is closely linked to peripheral muscle strength and respiratory function. Enhanced respiratory muscle strength improves vital capacity and respiratory efficiency, providing stable respiratory support during prolonged exercise (Akinoglu et al., 2019). However, in endurance-dominant sports (e.g., long-distance running, cycling), prolonged high-intensity exercise can induce respiratory muscle fatigue, which further impairs respiratory muscle strength, leading to severe fatigue and reduced exercise capacity (Loke et al., 1982; Ker and Schultz, 1996). Fortunately, research has shown that athletes’ respiratory muscles recover rapidly from fatigue, returning to baseline function within a reasonable recovery period (Ross et al., 2008).

### 4.3 Mechanisms underlying the effects of combined training

The present study’s findings align with previous research on IMT. IMT enhances respiratory muscle strength by stimulating adaptive remodeling of the inspiratory muscles (e.g., increased diaphragmatic cross-sectional area), which improves respiratory efficiency and reduces fatigue and dyspnea after strenuous exercise (Illi et al., 2012; Enright et al., 2006; Downey et al., 2007). During aerobic exercise, stronger respiratory muscles facilitate more efficient oxygen uptake and carbon dioxide elimination, thereby improving metabolic efficiency (Mcconnell and Romer, 2004).

Lower limb resistance training complements IMT by enhancing lower limb muscle strength and endurance, which increases systemic metabolic rate and athletic performance—both of which contribute to improved cardiopulmonary function (Clark et al., 2008). Lower limb muscles are the primary working muscles during most forms of exercise; increased lower limb strength directly enhances energy expenditure and oxygen utilization during exercise, thereby boosting cardiopulmonary function.

The significant increases in MIP, MEP, AT, and VO_2_max observed in both groups post-training (Figures 4, 5) are consistent with the findings of Brown et al., who reported that IMT significantly improves respiratory muscle strength and cardiopulmonary function (Brown et al., 2014). Other studies have also shown that IMT enhances VO_2_max and respiratory threshold, exerts positive effects on pulmonary function, and improves respiratory muscle endurance and coordination—all of which contribute to better respiratory stability and efficiency during prolonged exercise (Amonette and Dupler, 2002; Ogawa et al., 2020). A meta-analysis of respiratory muscle training in athletes further confirmed that targeted respiratory muscle training enhances respiratory muscle strength, vital capacity, and respiratory efficiency, leading to improved athletic performance (Hajghanbari et al., 2013).

The novel contribution of the present study lies in the integration of IMT and lower limb resistance training, which elicited synergistic effects. Compared to the control group, the experimental group showed significantly greater improvements in MIP, MEP, and VO_2_max after 12 weeks of training (Figures 4, 5). This aligns with previous research on combined training, which has shown that integrating different training modalities (e.g., aerobic + resistance, respiratory + core stability) more effectively enhances overall performance, particularly in endurance and strength (Jones et al., 2003; Smith et al., 1992). The combined training regimen in this study effectively increased respiratory muscle volume and function, leading to more efficient breathing.

### 4.4 Interpretation of static pulmonary function results

In contrast to the improvements in respiratory muscle strength and cardiopulmonary function, no significant changes in SVC, FVC, FEV_1_, or MVV were observed in either group post-training (Figure 6). This appears inconsistent with some previous studies, but this discrepancy can be attributed to differences in study populations. Most previous studies on respiratory muscle training and static pulmonary function have focused on patients with stroke or elderly individuals (Liu et al., 2022; Hu and Kai, 2022; Zhao et al., 2020), whereas the present study’s participants were military cadets with prior endurance training experience.

Research has shown that athletes have approximately 20% higher FEV_1_ values than sedentary individuals (Nottin et al., 2002). The cadets in this study had already undergone regular endurance training before enrollment, leading to adaptive changes in their respiratory systems (e.g., increased lung volume, enhanced respiratory muscle function). As a result, the additional training intervention (either IMT alone or combined training) had a minimal impact on static pulmonary function indices, which were already near their physiological ceiling (Klionsky et al., 2012).

### 4.5 Implications for military training

Respiratory muscle fatigue is a major limiting factor during high-intensity exercise. Targeted resistance training for respiratory muscles can delay or alleviate this fatigue, thereby improving exercise performance and endurance (Mcmahon et al., 2002). Incorporating respiratory muscle training modalities (e.g., resistance trainers, specific breathing exercises) into military training programs can enhance running performance and overall physical adaptability (Harms et al., 1985; Gething et al., 2004). While some researchers have suggested that combining inspiratory and expiratory muscle training may yield greater benefits, the present study demonstrates that combined IMT and lower limb resistance training is already an effective strategy for military personnel.

### 4.6 Limitations and future directions

Despite its contributions, the present study has several limitations:

Small sample size: The sample size of 60 participants may limit the generalizability of the results. Future studies should increase the sample size to improve statistical power.

Limited study population: The study focused exclusively on non-commissioned officer cadets from one military academy; results may not be applicable to other military branches (e.g., navy, air force) or personnel with different training backgrounds.

Short intervention duration: The 12-week training period only evaluates short-term effects; long-term outcomes (e.g., 6–12 months) remain unknown.

Future research should address these limitations by expanding the sample size, including diverse military populations, and extending the intervention duration. Additionally, exploring the mechanisms underlying the synergistic effects of combined training (e.g., molecular adaptations in respiratory and skeletal muscles) and optimizing the training protocol (e.g., adjusting intensity, frequency, or exercise order) could further improve the efficacy of military physical training programs.

## 5 Conclusion

The present study demonstrates that combined inspiratory muscle resistance training (IMT) and lower limb resistance training is more effective than IMT alone in enhancing cardiopulmonary function and respiratory muscle strength in military personnel. After 12 weeks of training, the experimental group (combined training) showed significantly greater improvements in VO_2_max and MIP compared to the control group (IMT alone).

Based on these findings, we recommend that military personnel incorporate respiratory muscle strength training—particularly combined IMT and lower limb resistance training—into their daily training programs to enhance physiological performance and athletic capability. This study provides a scientific basis for military physical training and offers an effective training strategy for other populations seeking to improve cardiopulmonary function and respiratory muscle strength.

Future research should validate and refine these findings using larger sample sizes, longer intervention periods, and more diverse study populations to provide evidence-based training guidance for multiple military branches.

## Data Availability

The original contributions presented in the study are included in the article/supplementary material, further inquiries can be directed to the corresponding authors.
